# EMT and Treatment Resistance in Pancreatic Cancer

**DOI:** 10.3390/cancers9090122

**Published:** 2017-09-12

**Authors:** Nicola Gaianigo, Davide Melisi, Carmine Carbone

**Affiliations:** 1 Digestive Molecular Clinical Oncology Research Unit, Section of Medical Oncology, Department of Medicine, University of Verona, Verona 37134, Italy; nicola.gaianigo@studenti.univr.it (N.G.); davide.melisi@univr.it (D.M.); 2 Medical Oncology Unit, Azienda Ospedaliera Universitaria Integrata, Verona 37134, Italy

**Keywords:** pancreatic cancer, EMT, resistance

## Abstract

Pancreatic cancer (PC) is the third leading cause of adult cancer mortality in the United States. The poor prognosis for patients with PC is mainly due to its aggressive course, the limited efficacy of active systemic treatments, and a metastatic behavior, demonstrated throughout the evolution of the disease. On average, 80% of patients with PC are diagnosed with metastatic disease, and the half of those who undergo surgery and adjuvant therapy develop liver metastasis within two years. Metastatic dissemination is an early event in PC and is mainly attributed to an evolutionary biological process called epithelial-to-mesenchymal transition (EMT). This innate mechanism could have a dual role during embryonic growth and organ differentiation, and in cancer progression, cancer stem cell intravasation, and metastasis settlement. Many of the molecular pathways decisive in EMT progression have been already unraveled, but little is known about the causes behind the induction of this mechanism. EMT is one of the most distinctive and critical features of PC, occurring even in the very first stages of tumor development. This is known as pancreatic intraepithelial neoplasia (PanIN) and leads to early dissemination, drug resistance, and unfavorable prognosis and survival. The intention of this review is to shed new light on the critical role assumed by EMT during PC progression, with a particular focus on its role in PC resistance.

## 1. Introduction

According to the American Cancer Society, pancreatic cancer (PC) is ahead of breast cancer as the third leading cause of cancer-related death in the United States, and is predicted to become the second leading cause of cancer-related death by 2020 [1,2]. Currently, PC has a distinctive adverse prognosis, with an overall five-year survival rate of <6%. This is primarily due to late diagnosis, which is aggravated by the absence of early recognizable symptoms in patients and by the lack of effective diagnostic and prognostic markers [3]. In the last two decades, development of new therapeutic drugs has been disappointingly stagnant. Indeed, since the late 1990s gemcitabine has represented the standard of care for advanced PC, although it does not show a drastic improvement in median survival rate [4]. This is mainly explained by the unique chemoresistance of PC cells [5].

Although the histology and the genetic of pancreatic carcinogenesis have been well described [6], the molecular mechanisms that promote the metastatic spread of PC are less clear [7]. These mechanisms include the ability of cancer cells to break away from extracellular matrix (ECM) and to overcome apoptosis process. This behavior has been associated with an early epithelial-to-mesenchymal transition (EMT) in premalignant lesions [8].

EMT is a well-coordinated process triggered by many signaling pathways during embryonic development, however it is also a pathological feature in neoplasia and fibrosis [9]. Cells undergoing EMT progressively lose the expression of components in the epithelial cell junctions. Instead, they produce a mesenchymal vimentin cytoskeleton and acquire both invasive and chemoresistance properties. Recent studies have also proposed that metastasis is an early event in the natural history of PC and could even precede tumor formation [10].

Thus, improving knowledge of molecular mechanisms that impair the response of cancer patients to chemotherapy is essential to designing more effective treatments for this deadly disease. In this review, we summarize the role of EMT in the context of drug resistance and metastasis in PC, with a special focus on inflammation.

## 2. EMT and Cancer Progression

EMT and the opposite process, mesenchymal-to-epithelial transition (MET), are innate and essential mechanisms implicated in cellular remodeling and tissue repair. The very first function of EMT occurs during gastrulation, when the blastomer differentiates into the three primordial cell lineages. EMT is constantly repeated out over the lifespan, from fetus development to tissue regeneration in adulthood [11]. It essentially relies on the plastic transformation of cells with an epithelial cobblestone phenotype, which are characterized by an apico-basal pattern of polarization and the establishment of tight junctions with the nearby cellular population, into cells showing distinctive mesenchymal features such as loss of three-dimensional organization in space, lack of cell polarity, and secretion of proteins constituting the backbone of extracellular matrix [12]. This same transition can be observed throughout the evolution of many tumors originating from epithelial cells; in fact, the progressive acquisition of gain and loss of mutations increases their chances of evading the solid tumor microenvironment, eventually resulting in metastasis formation and seeding in distant organs [13].

**Figure 1:**
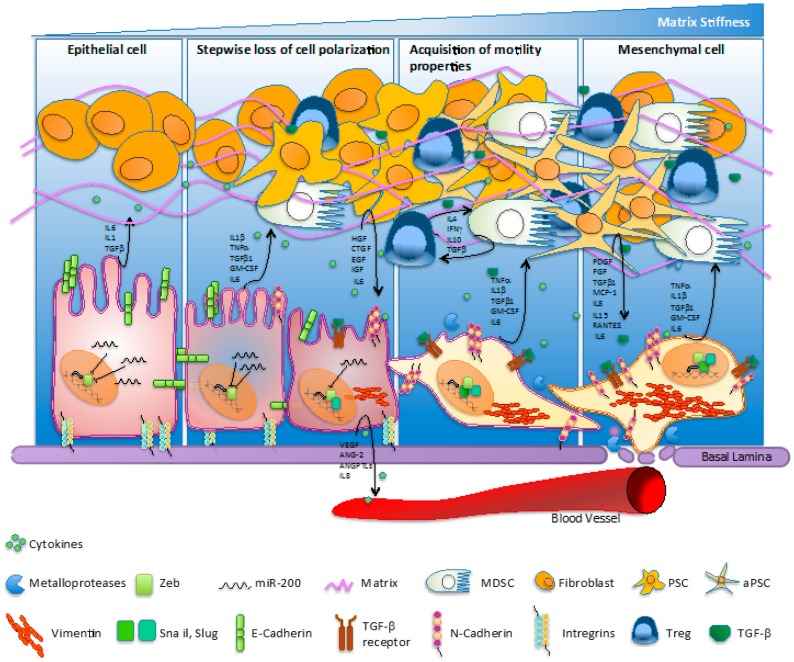
Molecular hallmarks/fluctuations/switching regulating the epithelial-to-mesenchymal transition (EMT) process in pancreatic cancer. The EMT process involves loss of cell polarization, a gain in migratory abilities and progressive acquisition of a mesenchymal phenotype. The EMT mechanism is characterized by the ‘cadherin switch’, where E-cadherin expression is progressively downregulated and replaced by the expression of N-cadherin. The transition process is associated to a decrease of miR-200 levels and an increase of classical E-cadherin transcriptional suppressors—such as ZEB1, Snail, and Slug—activated upstream by TGF-β. Cells undergoing EMT commonly quit the expression of extracellular matrix (ECM) elements mediating structural rigidity and cell adhesion in favor of proteases, cytokines, growth factors, and ECM components which improve cell migration and intravasation in bloodstream. Pancreatic cancer cell cytokines accelerate transformation of fibroblasts into quiescent pancreatic stellate cells (PSCs) and then into activated pancreatic stellate cells (aPSCs). Furthermore, inflammatory cytokines recruit myeloid progenitor cells and mediate their subsequent differentiation into myeloid-derived suppressive cells (MDSCs), which suppress the immune surveillance function. IL: interleukin; TGF: transforming growth factor; TNF: tumor necrosis factor; GM-CSF: granulocyte-macrophage colony-stimulating factor; HGF: hepatocyte growth factor; CTGF: connective tissue growth factor; EGF: epidermal growth factor; IFN: interferon; PDGF: platelet-derived growth factor; MCP-1: macrophage inflammatory protein 1; RANTES: regulated upon activation normally T-expressed and presumably secreted; VEGF: vascular endothelial growth factor; ANG-2: angiopoietin-2; ANGPTL: angiopoietin-like.

Many studies have determined which genes are regulated during the EMT process, comparing mesenchymal cancer cells and their epithelial counterparts [14,15]. Among these, downregulation of E-cadherin levels and activation of the transforming growth factor (TGF)-β-related signaling pathway are critical steps strictly required for EMT initiation and metastasis in PC [16,17] (Figure 1). This process involves the partial loss of cellular adhesive junctions, and progressive acquisition of typical mesenchymal markers, such as the neural-cadherin (N-cadherin or CH2) membrane protein (an event described as “cadherin switch”), as well as the expression of fibroblast-secreted extracellular proteins like type I collagene and some metalloproteases [18,19]. Notably, diverse kinase-dependent cellular signaling pathways—such as PI3K/AKT, EGFR, platelet-derived growth factor (PDGF), TAK1, and RAS—have been demonstrated to strongly affect E-cadherin subcellular localization and expression patterns [20,21]. Levels of E-cadherin are tightly regulated by the expression of its own suppressor (also called EMT-activating transcription factor, EMT-ATF), which are commonly subdivided into two main categories.

The first group is composed by SNAIL1, SNAIL2, ZEB1, ZEB2, E47, and KLF8 factors, which primarily act as repressors of *CDH1* promoter but also as down-regulators of genes implicated in maintaining cellular polarity, such as *LGL2*, *PATJ*, and *CRB3* [22,23].

The second group includes Twist, E2.2 and FoxC2 factors, which are responsible for *CDH1* transcription repression via indirect approaches and are principally induced under hypoxic conditions [24]. There is evidence to suggest that high levels of SNAIL and ZEB1 proteins are correlated with cancer disease relapse and short-term survival in many different typologies of cancer, highlighting how the EMT process might be one of the key reasons for dismal clinical outcomes in patients [25]. Interestingly, other EMT-driven mechanisms appear to strongly influence cancer progression at a deeper nuclear level. Biamonti et al. for instance, revealed how during the EMT process the CD44 transmembrane protein—a receptor for many proteins residing in the ECM including hyaluronic acid, osteoporin, collagene, and metalloproteases—undergoes selective alternative splicing, generating a mRNA isoform that differs from the “standard” epithelial variant [26]. To the same extent, the impact provided by re-modulation of the epigenetic landscape in cancer cells, both via methylation of target genes and histones modifications, has been intensely explored and classified as a crucial mechanism in EMT [27].

## 3. KRAS-Addiction of Pancreatic Cancer

Oncogenic KRAS plays a crucial role in the development of PC. Mutation of KRAS occurring in murine pancreas is sufficient to initiate acinar-to-ductal metaplasia (ADM) and pancreatic intraepithelial neoplasia (PanIN), which progress with long latency to invasive metastatic pancreatic ductal adenocarcinoma PDAC, thus recapitulating human disease [28,29].

Recently, a new mice model has been developed, called iKRAS, where pancreatic KRAS is placed under a tetracycline inducible promoter, providing a reliable and accurate model to trace the effects of KRAS contribution at different time points throughout cancer evolution [30]. Interestingly, expression of oncogenic KRAS in adult mice leads to the formation of PanIN lesions under circumstances of long latency and low penetrance, thus proving how a single mutation at KRAS oncogene is not sufficient to affect tissue organization and develop pancreatic neoplasia. At early checkpoints of PDAC evolution, KRAS-deregulated activity is strictly essential for PanIN progression, an effect that is observed also during late stage of tumor evolution, where neoplastic cells seem to undergo apoptosis upon KRAS oncogene inactivation [30].

These data support the idea of a KRAS addiction in which PC onset and evolution is essentially dependent on KRAS mutation. However, specific strategies aimed at KRAS targeting, such as disturbance of its membrane association, developing synthetic lethal interactions, and targeting of its downstream pathway or metabolic processes, showed few benefits in clinical practice [31].

Singh and colleagues, using an RNAi-based assay to deplete KRAS in a panel of KRAS-mutated PC cell lines, identified two classes of cells that do or do not require KRAS to maintain viability. The comparison between these two classes revealed a particular gene expression signature for KRAS-dependent cells, associated with a well-differentiated epithelial phenotype. They established that KRAS dependency is strongly linked to epithelial differentiation status, whereas most KRAS-independent cells appeared to assume a less epithelial phenotype [32].

It is widely accepted that poorly-differentiated tumors are more drug resistant and are associated with poor prognosis, highlighting a crucial role of the KRAS oncogene during the first steps of carcinogenesis of the independent KRAS cell lines [33].

Many fundamental cellular signaling cascades were investigated for their crucial involvement in tumor progression in KRAS-independent pancreatic cancer cell lines. Singh and colleagues hypothesized PI3K as the main driver of neoplastic phenotype in KRAS-independent cell lines [32]. Likewise, many studies supported the role of the nuclear transcription factor yes-associated protein 1 (YAP1) in compensating cancer cell survival and proliferation in KRAS-independent neoplasms, including PDAC [34,35]. The transcription factors YAP and TAZ, the main transducers of the Hippo pathway, have recently emerged for their association with pancreatic cancer ECM increasing stiffness [36]. YAP/TAZ are crucial downstream effectors of physical stimuli originating from the extra cellular environment surrounding tumor cells. ECM stiffness indeed promotes YAP/TAZ nuclear shuttling in cancer cells, leading to transcription of target genes regulating the EMT phenotype and chemoresistance [37].

## 4. Markers of EMT in Advanced Pancreatic Neoplasia

Roles and functions of EMT driving forces, described in the previous paragraph and normally upregulated in several cancers [38], were also studied in PC. In an immunohistochemistry analysis conducted by Hotz and colleagues in resected PC tissue samples, the expression of Snail and Slug factors accounted for a total of 80% and 50% respectively, whereas Twist showed little or no expression [39]. In a different study performed on 68 PC and 38 normal pancreas tissue samples, Twist nuclear levels were found to be decreased in tumor tissues, while staining of Slug or N-cadherin markers did not show a significant difference among healthy patients [40]. ZEB1 is considered one of the main inducers of EMT conversion in PC tissue in response to stimuli received from TGF-β and NF-κB pathways. Silencing of *ZEB1* in different PC cell lines resulted in a sensitive upregulation of epithelial markers and overall increased sensitivity of chemo-resistant cell lines to different chemotherapeutic agents, measured by an enhanced apoptotic response [41].

Loss of function mutations or downregulation of miRNA-200 family members, principally expressed by epithelial cells and identified as essential negative regulators of EMT and metastatic processes, were determined to indirectly stabilize ZEB1 expression levels and, in the meantime, to reduce E-cadherin expression in pancreatic β-cells, thus enhancing cells progression toward a mesenchymal phenotype [42]. The expression levels of miRNAs assume a tissue-specific pattern both in normal tissue and in PC and could function as potentially predictive diagnostic biomarkers. Downregulation of miR-148a and miR-217 and upregulation of miR-196a, miR-155, miR-203, miR-210, miR-222, and especially miR-21, were associated with a reduction of E-cadherin, an increase of vimentin levels, and with an overall poorer survival rate of patients [43,44]. Interestingly, miR-21 and miR-155 are both widely known as onco-mi-RNAs and their overexpression is linked to enhanced invasiveness, metastasis, and tumorigenesis onset in PC. This effect is thought to take action by compromising anti-inflammatory signaling pathways such as Ship1, or through the suppression of SOCS1 cytokine signaling [45] and the targeted modulation of peculiar tumor suppressor genes in tumor-supporting stromal cells [46].

PC is histologically recognized by an advanced fibrotic response, often referred as pancreatic desmoplasia, which is proved to confer the tumor microenvironment an increased resistance from many external stimuli including chemotherapeutic drugs, hormones, or cytotoxic mediators released by the host immune system [47]. Moreover, the discovery of a small subpopulation of cells with stem cell-like properties, termed cancer stem cells (CSCs), and residing within the tumor microenvironment, opens new possibilities for a more targeted therapy in several types of cancer. Pancreatic CSCs were initially isolated from a niche presenting the cell surface markers CD44 and epithelial-specific antigen (ESA). Many other markers of stemness—including CD133, CD24, c-Met, and CXCR4—were identified to selectively isolated PC stem cell (PCSC) population [48]. These special cells exhibit peculiar stem cell properties of self-renewal, including the ability to produce a differentiated progeny and an enhanced tumorigenic potential compared to control pancreatic cells [49]. Although the direct molecular correlation between EMT and CSC is still largely unknown, it is believed to rely on the similar pattern of activated signaling pathways, like Notch, Wnt/β-catenin, and the sonic hedgehog pathway (SHH), that are commonly shared in both.

## 5. EMT and Metastasis

Two main models of metastasis progression are hypothesized in the scientific community: a “Darwinian” linear model of evolution, which describes metastasis as a result of stepwise accumulation of genetic and epigenetic mutations that ultimately promote invasive behavior and dissemination during late tumor stage, and a parallel model of progression, where metastatic founder cells are believed to develop into circulating tumor cells (CTCs) and disseminate long before disease is clinically detected [50]. The early dissemination (parallel progression) model is dated back to the early 1950s and states that primary tumor and metastasis develop in parallel and acquire different genetic and epigenetic mutations throughout the progression. A recent mathematical modeling study, using the autopsy and the adjuvant cohort data from PC patient samples, predicted the presence of cells that are able to establish metastasis (not necessarily metastatic disease) even when the size of the primary tumor is still small [51,52]. More recently, in a pioneer study conducted by Rhim et al. [10], a sensitive method to tag and track pancreatic epithelial cells throughout cancer evolution was developed. Surprisingly, those cells were found to enter the bloodstream and seed in the liver even before the original cancer mass could be evidently detected through histologic analysis. In particular, they developed a Cre-Lox based mouse model of PC (called PKCY). In 8–10-week-old PKCY mice, only PanIN lesions were present, and 2.7% and 6.8% of PanIN 2 and 3 lesions, respectively, showed at least one YFP^+^ ZEB1^+^ cell on staining. Among these, they identified single YFP^+^ cells that had crossed the basal lamina and had started to acquire a mesenchymal-like phenotype, making them almost identical to surrounding stromal cells. Surprisingly, cytofluorimetric analysis identified circulating pancreatic cells (CPCs) in the bloodstream of 8–10-week-old PKCY mice; moreover liver seeding of YFP^+^ cells was detected in 4 out of 11 PanIN mice, although most of them were single cells located near blood vessels but with no evident expression of ZEB1. Overall, these data support a model for PC progression in which metastasis seeding in distant organs occurs before/in parallel to tumor development at the origin site. In another pilot study by Yu et al. [53], RNA sequencing analysis on circulating pancreatic tumor cells (CPCs) originating from genetically engineered mouse models of PC showed a pronounced enrichment for *WNT*2 gene, a member of non-canonical WNT pathway, compared to primary tumor cells. Expression of *WNT2* in PC cells suppresses anoikis, enhances anchorage-independent growth of spheres, and increases metastatic propensity in vivo. Moreover, an upregulation for *WNT* genes was also confirmed in the CPCs directly isolated from PC patients. Conversely, Zheng et al. [54] proposed a study where two engineered (KrasLSL.G12D/+; p53R172H/+; PdxCretg/+) (KPC) mouse models were created, one carrying a deletion in Twist1 and the other in Snail1 genes. Unexpectedly, PC evolvement and metastasis formation were not prevented by the absence of the two principal genes involved in EMT progression; indeed, metastasis occurred both in lung and liver. Moreover, engineered mice developed PanIN lesions with the same frequency as normal KPC mice, despite exhibiting a significant loss in the EMT course. Hypothetically, this behavior might be due to a partial balance effect provided by other cellular factors involved in EMT. However, resistance to gemcitabine was significantly lower in the two mouse models. This is the first study claiming that EMT is a side process that is significant for cancer progression and metastasis evolution but not rate-limiting as universally believed [54].

## 6. Molecular Mechanisms of EMT and PC Treatment Resistance

Emerging evidence suggests a molecular and phenotypic association between increased chemoresistance and gaining of EMT-like phenotype of cancer cells. Different papers demonstrated that PC cells treated with an increasing regimen of chemotherapy drugs developed early resistance. A genome-wide array of the most differential expressed genes among the chemoresistant and chemosensitive PC cell lines indicated a distinctive connection with genes participating in the EMT process. RT-PCR analysis confirmed high levels of ZEB1 and vimentin in the chemo-resistant cells specifically, while target silencing of ZEB1 restored E-cadherin expression and led to an overall increased drug sensitivity [41]. The same effects were achieved when two pancreatic cell lines, L3.6pl and AsPC-1, were exposed to increasing concentration of gemcitabine until they developed an intrinsic resistance. These cells demonstrated a marked loss of cell-cell adhesion, formation of pseudopodia and enhanced mesenchymal-like morphology. Migration and invasion rates were also strongly enhanced in gemcitabine-resistant (GR) cells. Moreover, proteins such as β-catenin, E-cadherin, and vimentin, which are among the principal hallmarks of EMT, were found to radically relocalize into GR cells in a pattern consistent with the gaining of mesenchymal features [55]. Flow cytometry data proved also that the GR cell population carried increased cancer stem cell markers, including CD24, CD44, and ESA. Therefore, it has been hypothesized that the impressive resistance of PC to standard chemotherapy and radiation treatment could be owed to the presence of CSCs, which are documented to express high levels of multidrug-resistant membrane transporters, and abnormally activated signaling cascades promoting cell proliferation, migration and invasion.

Recently, the protein expression of the stem cell marker Nestin has been used to identify progenitor cells in several tissues and was investigated its association with tumor staging and metastasis formation in several cancers comprising PC [56]. Nestin has been linked with pancreas development and PC progression. Moreover, it was demonstrated that the activation of oncogenic KRAS in Nestin^+^ cell lineage is sufficient to drive the initiation of PanIN lesions in pancreatic tissue [57]. TGF-β1 was proved to induce the expression of Nestin in PDAC cells, predominantly through the SMAD4 pathway. At the same time, overexpression of Nestin itself leads to an autocrine feedback loop triggering the production of TGF-β1 and its receptors both at RNA and protein levels [56]. Efforts were also dedicated in studying the expression pattern of the membrane receptor tyrosine kinase c-KIT (CD117) and its relative ligand, the stem cell factor (SCF), both in normal tissue and in advanced PC. c-KIT expression is normally restricted to the embryonic and fetal life of humans, and to date only few studies suggest its correlation with cancer progression and dissemination, although the data retrieved so far are partially conflicting. Immunohistochemistry tests performed on normal human adult pancreas and on pancreatic ductal adenocarcinoma, revealed that the expression of c-KIT protein is enhanced in the latter, probably due to mutations in its aminoacidic sequence, and seem to be initiated in β-cells of the islets of Langerhans, progressively spreading toward the cancerous ducts. Immunostaining of pancreatic metastasis in liver displayed localization of the c-KIT protein both in the cell membrane and within the cytosol, while its expression was considerably increased compared to the original PC tissue [58]. Wang et al. [59] confirmed that vimentin, α-smooth muscle actin (α-SMA), and ZEB1 are strongly upregulated in gemcitabine-resistant pancreatic cells compared to normal epithelial cells and demonstrated that Notch signaling is one of the leading pathways driving EMT process. RNA and protein levels for Notch pathway were shown to have an increased activation of Notch-2, Notch-4, and Jagged-1 in GR cells [59]. At the same time, expression of NF-κB, one of the principal downstream targets of the activated Notch pathway and the central mediator of EMT in cancer progression, was found to be considerably upregulated. SMAD4 protein is also altered or inactivated in the majority of PC cases, an event that is normally associated with a simultaneous inactivation of INK4A/ARF tumor suppressor gene and activation of KRAS oncogene. In vivo experiments conducted on genetically engineered mice carrying deletion on the *SMAD4* gene showed that the selective knock out of this signal transducer did not influence the physiology of pancreas organ. Interestingly, when combined with *KRAS* activating mutation, SMAD4 deficiency allowed a first rapid evolution of the early stages of pancreatic lesions toward mature PC, but this progression was not combined with further dissemination. *SMAD4*-deficient invasive tumors retained a differentiated ductal histopathology, confirmed by the expression of the E-cadherin epithelial marker [60].

The most recent and advanced targeted therapy showed a slight increment of efficacy in PC, with gemcitabine remaining the most largely used chemotherapeutic drug in PC. This is mainly due to the lack of specific druggable targets and to the substantial resistance that commonly arises along treatments [61].

Constitutive activation of NF-κB in PC represents the main intrinsic mechanisms of resistance due to suppression of apoptosis mechanisms. Experimental pre-clinical evidence supports the potential efficacy of anti-NF-κB strategies, but to date there are no direct NF-κB inhibitors available for patients [62]. However, the possibility of inhibiting NF-κB seems to be achieved indirectly by using inhibitors of specific mediators required for NF-κB activation [63]. In this regard, the use of a TAK1 inhibitor has successfully proven to overcome chemoresistance in PC [64], as well as esophageal cancer models [65].

Pancreatic cells expressing EMT markers also showed an increased resistance to the EGFR inhibitor [66]. The concomitant inhibition of NEH1, a molecular partner of EGFR, with erlotinib results in a decreased three-dimensional colony growth and invasion for both classical and mesenchymal PC cell lines [67].

Another successful approach is the use of monoclonal antibodies against EGFR, cetuximab, and panitumumab, which inhibit receptor dimerization at the extracellular domain and significantly increases radiosensitivity in locally advanced PC. However, the gemcitabine/erlotinib combination also results in an increased toxicities and higher relative cost compared to gemcitabine alone, which are important factors to discuss with patients when reviewing their therapeutic options [68]. In addition, current studies showed that an anti-vascular endothelial growth factor (VEGF) treatment could induce a significantly more aggressive and metastatic phenotype of tumor cells. However, the molecular mechanisms and mediators behind this phenomenon are still unrevealed.

It is increasingly clear that angiogenesis enhances metastatic potential and promotes progression, thus impairing the angiogenic potential of PC, through anti-VEGF therapy, could improve the efficacy of chemotherapy [69]. Recently, in a model of preneoplastic lesions, we showed that autocrine signaling of ANGPTL2 and its receptor, LILRB2, plays key roles in sustaining EMT and the early metastatic behavior of cells in two models of pancreatic preneoplastic lesions [70].

Recently, our group established the tumor cell-initiated mechanisms responsible for the resistance of PC to anti-VEGF treatment. We identified several proinflammatory factors that were expressed at higher levels in cells resistant to anti-VEGF treatment than in treatment-sensitive control cells. These proinflammatory factors acted in a paracrine manner recruiting CD11b^+^ proangiogenic myeloid cells and thus inducing EMT [71]. More recently we demonstrated that combined inhibition of proinflammatory interleukin (IL) 1, CXCR1/2, and TGF-β signaling pathways might reverse this anti-VEGF resistance, reversing epithelial–mesenchymal transition and inhibiting CD11b^+^ proangiogenic myeloid cells’ tumor infiltration [72]. We integrate the secreted factor responsible of anti-VEGF resistance into the single transcription factor HOXB9. Since the silencing of HOXB9 modulates the anti-VEGF resistance in both pancreatic and colorectal cancer cells, we propose HOXB9 as a candidate predictive biomarker for selecting cancer patients for antiangiogenic therapy [73].

Extrinsic regulators of chemoresistance are represented by cellular components of the stroma, and the extracellular matrix that they produce. Abundant desmoplastic stroma may represent a barrier for chemotherapetic drugs delivering to the tumor. However, approaches to inhibiting the signaling pathways that regulate collagen secretion by fibroblast, such as SHH signaling inhibitors, have not reached the advanced stage of clinical trials so far [74].

## 7. Role of Desmoplasia in Pancreatic Cancer

PDAC is characterized by a prominent stromal-desmoplasmic reaction [75]. This desmoplasmic/fibrotic state is commonly promoted by the tumor microenvironment itself and is usually characterized by an abundant deposition of structural proteins, an altered organization, increased secretion of growth factors, and enhanced post-translational modifications (PTMs) of ECM proteins [76]. The PDAC stroma is a highly complex structure composed by several extracellular molecules, such as collagen (mainly type I), vitronectin, fibronectin, hyaluronic acid, molecular growth factors, and several specialized cells including endothelial cells (blood vessels), neural cells, cancer stem cells, immune cells, pancreatic stellate cells (PSCs), and cancer-associated fibroblasts (CAFs) [74,77].

Usually, cancer treatment failings are mainly attributed to the stroma enflaming chemoresistance, as well as decreasing microvascularity and, therefore, reduced drug delivery in the PDAC environment [78,79,80]. Stroma contribution to cancer advancement has been a fundamental subject of study in recent years, an issue that granted tumor-associated extracellular matrix to be listed as one of the main hallmark of cancer disease. This new field of research has shed new expectation for the development of novel therapeutic approaches to overcome tumor–ECM crosstalk and disease progression, however the molecular mechanisms regulating the fine balance between pancreatic carcinoma and desmoplasia are still largely unclear [47].

In normal pancreas, PSCs exist in a very low amount and in quiescent phase, but during carcinogenesis they slowly progress toward an activated phase, when they are referred as activated-PSCs (aPSCs). Similarly, CAFs can arise from PSCs, from residing activated fibroblasts or from close epithelial cells that underwent EMT. CAFs and aPSCs assume a myofibroblast-like phenotype and are mainly responsible for the pancreatic desmoplasmic reaction, through the expression and secretion of α-smooth muscle actin (α-SMA) and other proteins including fibroblast activation protein (FAP) and fibroblast-specific protein (FSP) [81].

Myofibroblasts specifically interact with cancer cells to create a tumor-promoting environment that stimulates resident tumor growth, treatment resistance, and metastasis formation. Their activation is dependent on several extracellular factors secreted by tumor itself, such as PDGF, TGF-β, TNFα, IL-1β and 6, cytokines that are in turn released by aPSCs and CAFs, triggering positive paracrine loops that sustain cancer progression [82,83]. Activation of myofibroblasts and infiltration of immune cells in tumor ECM environment dramatically increase matrix stiffness and stimulate cytoskeletal contractility of transformed epithelial cells, events that further contribute to amplify growth factor and cytokine signaling pathways. This stiffness increment promotes the formation of invadosomes, structures responsible for the production of metalloproteases required to demolish the ECM and to invade the basement membrane by driving focal adhesion assembly [84]. In a study conducted by Laklai et al. [85], using in vivo models they observed that PDAC showing impaired TGF-β activation signaling, increased β-catenin and YAP/TAZ nuclear localization and activity, developed a stiffer, fibrotic matrix associated with a more aggressive tumor and a poorer overall survival. Recapitulating, ECM stiffness directly fosters tumor malignancy and metastasis formation by promoting integrin-dependent matrix adhesion and invasion and regulating tumor plasticity [85].

The ECM is one of the main factors driving tumor resistance onset to many traditional chemotherapeutic treatments. For this reason, recently considerable clinical research has moved toward novel potential therapeutic approaches aiming to selectively targeting the tumor ECM environment rather than focusing solely on neoplastic cells. In particular, such trials have focused on targeting specific signaling molecules or remodeling enzymes, such as sonic hedgehog pathway (SHH), focal adhesion kinase (FAK) protein, and hyaluronidase inhibitors through neoadjuvant therapy [76,86]. The main outcome obtained in PDAC-ECM therapy however was the failure of SHH-targeted treatment, because despite the initial enthusiasm and success achieved by this therapy in preclinical studies, in in vivo SHH-deficient models, tumors showed a reduction in stroma content, but surprisingly, they also showed an increased vascularity, more invasive features, and a more aggressive phenotype [87].

## 8. Inflammation Sustains EMT in Pancreatic Cancer

PDAC is characterized by a large fibrotic tumor microenvironment, called desmoplasia, that hosts many different cell types including immune cells such as regulatory T cells (Tregs), tumor associated macrophages (TAMs), myeloid-derived suppressive cells (MDSCs), and natural killers (NKs) that support tumor growth, immune suppression and vascular growth (Figure 1). More specifically, PC exhibits a high abundance of regulatory CD4^+^ CD25^+^ FoxP3^+^ [88] and cytotoxic CD8^+^ T lymphocytes that linger near tumor boundaries. Despite the fact that both innate and adaptive immune responses are active against cancers, PC by itself induces local and systemic immune dysfunction in order to evade the recognition of cancer cells by activated immune effector cells [89].

Many of such infiltrated immune cells sustain mesenchymal transition of pancreatic cancer cells fueled by a local cytokine storm. For instance, TAMs have been described as potent EMT inducers through the secretion of several growth factors (hepatocyte growth factor (HGF), epidermal growth factor (EGF), TGF-β, PDGF, etc.) and inflammatory interleukins and cytokines (IL-1β, IL-6, and tumor necrosis factor (TNF)-α) that promote and support EMT. Interestingly, in vitro experiments demonstrated that the coculture of several PC cell lines with M2-polarized macrophage cells induced the acquisition of EMT properties in cancer cells [90].

MDSCs participate in the cytokine storm, secreting growth factors such as CCL2, TGF-β, and IL28, and inducing EMT in cancer cells [91]. Similarly, Th1 cells have been documented to activate innate immune cells, such as macrophages, and modulate the function of B cells and CD8^+^ T cells through cytokine secretion and direct cell-cell signaling [88]. Helper T cells are further differentiated in two interchangeable subtypes, Th1 and Th2, with the former principally implicated in cell-mediated immune response by secreting INF-γ and IL2. The latter are predominantly recruited in humoral immune responses, although several studies support their pro tumor-tolerance activity [92]. The cytokine storm in the tumor microenvironment converges mainly on the constitutive activation of NF-κB pathways, implicated in inflammatory-driven EMT intensification, tumor proliferation, and cancer resistance. As a downstream effect, tumor cells express molecules that further supply the inflamed environment such as granulocyte-macrophage colony-stimulating factor (GM-CSF), IDO, IL8, and most importantly TGF-β, IL-17, PDL-1, and FASL, increasing the immunosuppressive state of the tumor environment [93].

In order to evade immuno-surveillance, PC cells express non-functional FAS receptors (members of the TNF receptor family) or increase the secretion of functional FAS ligands, which promote the apoptosis of surrounding cancer infiltrating macrophages, dendritic cells, T lymphocytes and NK cells [94]. Moreover, released TGF-β also exerts an inhibitory function on CD8^+^ cytotoxic cells by blocking the expression of genes encoding cytolytic proteins, such as granzyme and perforin [95].

Thus, tailored immunotherapy is believed to represent the future of pancreatic cancer treatments and several clinical trials ongoing for pancreatic cancer disease involving the assessment of novel immune checkpoint inhibitors [96]. Immunotherapy efforts have recently focused in particular on three of the most important immune checkpoint participating in PDAC evolution: the cytotoxic T lymphocyte antigen 4 (CTLA-4), programmed cell death 1 (PD-1), and its ligand (PDL-1) [97,98].

CTLA-4 is an antigen expressed on Tregs and on activated CD4^+^ CD8^+^ T cells, and its activation modulates the suppression of immune responses by Tregs and effector T cells. Ipilimumab and Tremelimumab are two human therapeutic monoclonal antibodies developed to counteract CTLA-4 effects in cancer [99]. Unfortunately, CTLA-4 inhibition in pancreatic cancer through immunotherapy alone or in combination with standard chemotherapy has shown little or no efficacy so far, an effect probably due to the low number of immune cells residing in the PDAC microenvironment [100,101].

PD-1 is an immune checkpoint antigen expressed on activated dendrytic cells (DCs), monocytes, NK cells, and T and B cells. PD-1 selectively binds to antigen-presenting cells (APCs) programmed death ligands PD-L1/2, and to tumor cell ligand PD-L1 [102]. Interaction brings an anergy of effector T cells through the inhibition of many T cells downstream kinases and a reduction of IL-2 and INF-γ secretion. Surprisingly, PDAC and CAFs progressively acquire a potent immune resistance toward effector T cells by the expression of PD-L1, an action that both increases Treg infiltration in tumor microenvironment and induces T-cells inhibition, apoptosis and clearance [103]. Among the drugs designed to hinder the PD-1/PD-L1 interaction currently tested in ongoing clinical trials, there are the humanized monoclonal antibodies nivolumab, pembrolizumab, and pidilizumab, all targeting the PD-1 receptor, and durvalumab, which targets PD-L1 [97,104].

Even in this case, immunotherapy has shown poor results in pancreatic cancer. The only exception is represented by the monoclonal antibody pembrolizumab, which has been recently licensed by the Food and Drug Adminostartion (FDA) in an unprecedented early case of approval, for patients with advanced solid adenocarcinoma, including PDAC, showing mismatch repair defects [105].

In a revolutionary phase II clinical trial, Le and colleagues, evaluated the efficacy of pembrolizumab in a cohort of 41 patients with advanced metastatic carcinoma, with or without mismatch repair defects. Specifically, they hypothesized that the mismatched repair-deficiency mechanisms (MMR) were responsible for the positive responses of patients treated with this mAb. This type of genome instability is known to harbor thousands of somatic mutations, and occurs only on a small fraction of advanced colorectal tumors, characterized also by a prominent lymphocyte infiltrate. The efficacy of pembrolizumab was confirmed in a cohort of both colorectal and non-colorectal cancer patients mutated for MMR, thus representing the first drug based on a predictive cancer marker rather than a tumor type [105].

## 9. Microbiota, EMT, and Treatment Resistance

In recent years, accumulating evidence indicates that a microbiome alteration influences metabolism, tissue development, inflammation, and immunity of several tumors [106]. The gut microbiota influences both local and systemic inflammation [107], raising the question of whether the microbiota affects inflammatory processes that contribute to cancer progression and its therapy. To date, only few epidemiological studies have examined the association between microbiota or oral microbiota and risk of pancreatic cancer; however, the results from these studies are still controversial [108]. Diverse microbiome alterations exist among several body sites, including pancreatic tissue [109]. However, the role of the microbiome in EMT induction in pancreatic cancer cells is plausible but not yet clarified. The innate immunity is exceeded by microbe infection leading to chronic inflammation and activation of signaling pathways involved in EMT. Thus, growth factors and microbes share common signaling pathways, suggesting that microbes may be considered as EMT inducers [110]. Indeed, the most intriguing results regarding pancreatic cancer treatment resistance and microbiota have been published recently.

Accumulating evidence suggests that specific microbiome and microbial dysbiosis can potentiate both hepatobiliary and pancreatic tumor development by damaging DNA, activating oncogenic signaling pathways, and producing tumor-promoting metabolites [111].

Emerging evidence from mice models suggests that oral administration of microbiota may influence the efficacy of cancer chemotherapies and novel targeted immunotherapies such as anti-CTLA4 and anti-CD274 therapies, improving the function of tumor-specific CD8^+^ T cells [112,113].

Recently, Iida et al. demonstrated that optimal responses to cancer therapy require an intact commensal microbiota that mediates its effects by modulating myeloid-derived cell functions in the tumor microenvironment [114].

A recent randomized phase II POC study (IMAGE-1), combining heat-killed *Mycobacterium obuense* (IMM-101) with gemcitabine, suggests a beneficial effect on survival in patients with metastatic PDAC [115]. In a randomized study, Le and colleagues demonstrated that granulocyte-macrophage colony-stimulating factor (GM-CSF)-secreting allogeneic PDA cell lines (GVAX) followed by live-attenuated *Listeria monocytogenes*-expressing mesothelin, (CRS-207) significantly improved OS as compared with GVAX alone in patients with metastatic PDAC [116].

Novel functional analysis of patient-derived microbioma paired with preclinical models will enable the development of new types of anticancer therapy and could improve clinical intervention.

## 10. Conclusions

Increasing evidences suggest that EMT plays fundamental roles in cancer progression and resistance through several possible mechanisms, leading to a dramatic increase in disease aggressiveness, poorer disease elimination and overall patient survival.

Therefore, a thorough understanding of the underlying molecular features driving PC evolution is of utmost importance in order to develop effective therapies toward the original tumor, but also toward the population of cells responsible for drug resistance and metastasis formation. Moreover, the high inflammatory status and the complex network of immune cells recruited within advanced pancreatic tumor environment positively promote cancer progression and EMT transition of primary cancer cells rather than destroying malignant cells. Thus, new strategies targeting the EMT phenotype could increase sensitivity to both standard and targeted therapies and could improve the outcome of patients with PC.
